# Disrupted Functional Brain Connectivity and Its Association to Structural Connectivity in Amnestic Mild Cognitive Impairment and Alzheimer’s Disease

**DOI:** 10.1371/journal.pone.0096505

**Published:** 2014-05-07

**Authors:** Yu Sun, Qihua Yin, Rong Fang, Xiaoxiao Yan, Ying Wang, Anastasios Bezerianos, Huidong Tang, Fei Miao, Junfeng Sun

**Affiliations:** 1 Singapore Institute for Neurotechnology (SINAPSE), Center for Life Sciences, National University of Singapore, Singapore; 2 School of Biomedical Engineering, Shanghai Jiao Tong University, Shanghai, China; 3 Department of Radiology, Ruijin Hospital, Shanghai Jiao Tong University School of Medicine, Shanghai, China; 4 Department of Neurology, Ruijin Hospital, Shanghai Jiao Tong University School of Medicine, Shanghai, China; Institute of Automation, Chinese Academy of Sciences, China

## Abstract

Although anomalies in the topological architecture of whole-brain connectivity have been found to be associated with Alzheimer’s disease (AD), our understanding about the progression of AD in a functional connectivity (FC) perspective is still rudimentary and few study has explored the function-structure relations in brain networks of AD patients. By using resting-state functional MRI (fMRI), this study firstly investigated organizational alternations in FC networks in 12 AD patients, 15 amnestic mild cognitive impairment (aMCI) patients, and 14 age-matched healthy aging subjects and found that all three groups exhibit economical small-world network properties. Nonetheless, we found a decline of the optimal architecture in the progression of AD, represented by a more localized modular organization with less efficient local information transfer. Our results also show that aMCI forms a boundary between normal aging and AD and represents a functional continuum between healthy aging and the earliest signs of dementia. Moreover, we revealed a dissociated relationship between the overall FC and structural connectivity (SC) in AD patients. In this study, diffusion tensor imaging tractography was used to map the structural network of the same individuals. The decreased FC-SC coupling may be indicative of more stringent and less dynamic brain function in AD patients. Our findings provided insightful implications for understanding the pathophysiological mechanisms of brain dysfunctions in aMCI and AD patients and demonstrated that functional disorders can be characterized by multimodal neuroimaging-based metrics.

## Introduction

Alzheimer’s disease (AD) is a neurodegenerative disease clinically characterized by progressive dementia and neuropsychiatric symptoms include confusion, aggression, language breakdown and loss of cognitive functions. Mild cognitive impairment (MCI), characterized by memory impairment, is believed to be a transitional period between normal aging and AD. As a subclass of MCI, amnestic MCI (aMCI) is a syndrome with cognitive decline greater than expected for an individual’s age and educational level yet not fulfilling the criteria of AD. Subject with aMCI has a high probability (approximately 10%–15% per year) of evolving toward AD, up to 80% of these individuals would progress to dementia after 6 years [1]. Facing these serious facts, an upsurge of interest has been directed toward understanding the pathogenesis of AD and developing both diagnostic and prognostic biomarkers with a goal of predicting which individuals are more likely to progress and ultimately discovering effective therapies. Though previous studies showed the earliest regions affected by AD pathology are the transentorhinal cortex, parahippocampal gyrus, cingulum and hippocampal formation [2], [3], what are the effects of these focal damage on whole neural networks is still largely unexplored.

The human brain forms a large-scale network of interconnected brain regions which coordinate brain activities. Recent advances in neuroimaging techniques and graph theory methods allow for the investigation of human brain networks from topological perspective (for a review, see [4]). Accumulated studies of healthy populations have shown that human brain networks have special topological organization, including small-worldness, existence of highly connected network hubs, and modularity [5], [6]. Changes in brain network topology have been related to normal cognitive development and to a wide range of brain diseases, indicating a close relation between connectivity and cognitive status. For patients with AD, there is a growing body of studies suggesting that the cognitive dysfunction may result from abnormal wiring of the brain’s network (for a review, see [7]). Particularly, convergent evidence of a loss of the small-world network, an optimal brain network architecture characterized by high efficiency of information transfer with low wiring cost, has been revealed in AD [8]–[12]. Although small-worldness summarizes key properties of complex networks at both global and local levels of topological description, the brain network organization of AD patients at meso-level, which could be described by the modularity of network, is still limitedly understood.

Modularity is considered to be one of the main organizing principles in brain network [4], [13] and represents an optimal partition of a brain network into smaller functional communities or modules [4], [14], [15]. Each module contains subsets of densely interconnected nodes which are sparsely connected to nodes in other modules [14]. Theoretically, there are several advantages of modular brain network architecture. It provides an optimal solution for balancing the opposing demands that are placed on many dynamical systems: a high level of local specialization, while maintaining tight global integration [4]. Another major advantage of modularity is that it allows the brain to adapt to multiple or distinct selection criteria over time [16]: a modular-organized network can evolve or grow one module while maintain the functionality of other modules. Several previous studies have demonstrated the evolution of modular organization in the human during infancy [17], [18] and aging [19]–[21]. For instance, Chen and colleagues have demonstrated that elderly adults had less number of modules and significantly reduced modularity in anatomical brain networks when compared to young adults, which might be induced by the reduced functional segregation in the aging brain [22]. In the context of the recent focus on the developmental phenotypes of neuropsychiatric disease [23]–[25], the application of modularity can help us to reveal sensitive markers of abnormal brain development in brain diseases such as aMCI and AD. In fact, using resting state magnetoencephalography (MEG) signals, a dysmodularity of AD has recently been reported with a significant weakening of intermodular connectivity, indicating the loss of communication between brain systems that are specialized to carry out different cognitive tasks [25]. This study provides insightful implications on the understanding of how the modularity altered in aMCI and AD and inspires us to investigate how functional modular organization evolves from aMCI to AD.

There are widespread atrophy and abnormal structural connectivity in AD-related pathological regions, which raise another open problem in brain networks of AD, that is, how the abnormality of structural connectivity would affect the corresponding functional connectivity. Because the propensity for two populations to interact should vary, at least in part, to the density and efficacy of the projections connecting them [26]. In other words, connectivity of brain activity is predicted to be confined towards pathways of neuroanatomical connections between specific brain regions. Thus it makes sense to assume that the repertoire of functional configurations and interactions is reflective of underlying anatomical linkage [27], [28]. The emerging field of combining both functional and structural brain networks has provided some of the first quantitative insights to better understand brain dysfunctions associated with neurological and psychiatric disorders, which would significantly advance our diagnostic and prognostic capacities (for a review, see [29], [30]). Recently, Van de Heuvel et al., have revealed an increase in the strength of functional connectivity (FC) - structural connectivity (SC) coupling and speculate that it may be an indicative biomarker of less dynamic brain function in patients with schizophrenia [31]. Several attempts have also been made to uncover the AD related network changes with a combined approach of different imaging modalities, e.g., fMRI and DTI [32], [33]. However, in these pioneering studies, the comparisons of FC and SC are typically conducted in default mode network (DMN) regions; while the relationship between FC and SC evolves from NC to AD at the whole brain level is only beginning to be revealed.

**Figure 1:**
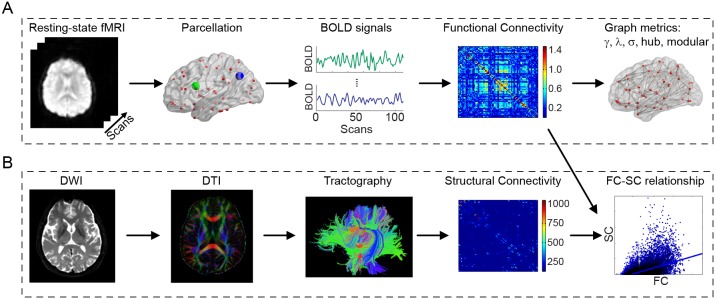
The flowchart of the analysis steps. (A) The construction of resting-state functional connectivity (FC) network for graph theoretic analysis. Note that, two parcellation scales (low: 90 ROIs, and high: 1024 ROIs) were employed to estimate the graph metrics. For better presentation purpose, only low parcellation scale was utilized here. (B) The construction of structural connectivity (SC) network for assessing the FC-SC relationship. In the current study, the level of coupling between FC and SC was examined by calculating the level of correlation between the weights of existing SC and their functional counterparts.

With above considerations, we constructed the functional brain network based on resting state fMRI data measured from healthy controls, aMCI and AD patients, and further investigated the topological characteristics of the brain networks by graph theoretical analysis (Figure 1A). We sought to determine whether functional brain networks would show altered topological organization in patients with aMCI and AD. Our hypothesis is that cognitive impairment in aMCI and AD will be reflected by the abnormal functional brain network characterizing of: *i*) a loss of small-world architecture, *ii*) an altered nodal betweenness centrality, and *iii*) a reduced modularity as well as a reduction in the connectivity of the modules. In network terms, we expect AD to be a ‘disconnection disease’. We further measured the SC using diffusion tensor imaging (DTI) tractography in the same individuals to investigate function-structure relations in brain networks of AD and aMCI patients (Figure 1B). We hypothesize that an altered relationship of functional-structural connectivity (FC-SC) would appear in aMCI and AD patients compared with healthy controls. We believe that this study would provide new insights on the understanding of AD development from the aspects of brain networks, especially on the alternation of modularity at meso-level and on FC-SC relationship in AD via multi-modal neuroimaging.

## Results

### 2.1 Demographic and Clinical Data

The demographic data and neuropsychological characteristics are shown in Table 1. Subject groups did not differ significantly in age (*p* = 0.348), gender distribution (*p* = 0.871), or years of education (*p* = 0.258). For the neuropsychological tests, there were significant between-group differences in MMSE scores (*p*<1.0×10^−5^), CDR (*p*<1.0×10^−4^) and ADL (*p*<1.0×10^−5^). *Post hoc* comparisons showed significantly reduced MMSE, CDR and ADL scores in both aMCI and AD patients relative to the normal controls.

**Table 1:** Demographic and neuropsychological data.

	NC	aMCI	AD	F value (χ^2^)	*p* value
Age (years)	68.64±8.36	70.20±6.94	73.08±7.90	1.09	0.348
Education (years)	12.86±3.61	10.87±4.88	9.92±5.25	1.41	0.258
Gender (M/F)	6/8	7/8	4/8	0.28	0.871^#^
MMSE	28.57±1.16	26.47±3.44	17.42±4.46	42.16	**<0.0001** * **^,^** † **^,^** ‡
CDR	0.29±0.26	0.53±0.13	1.50±0.67	32.63	**<0.0001** * **^,^** † **^,^** ‡
ADL	14.21±0.56	14.47±1.13	30.25±9.20	42.85	**<0.0001** † **^,^** ‡
ACER	86.57±6.91	75.47±11.87	39.33±13.79	62.88	**<0.0001** * **^,^** † **^,^** ‡

Values are represented as the mean ± SD. ^#^ *p* value for gender distribution in the three groups was obtained via a χ^2^ test. Separate ANOVA analysis was performed to investigate the group effect of age and neuropsychological data. NC: healthy controls, aMCI: amnestic mild cognitive impairment, AD: Alzheimer’s disease, MMSE: Mini-Mental Screening Examination, CDR: Clinical Dementia Rating, ADL: Activities of Daily Living, ACER: Addenbrooke’s Cognitive Examination Revised.

*Post hoc test showed significant group difference between NC *v.s.* aMCI;

† *Post hoc* test showed significant group difference between NC *v.s.* AD;

‡ *Post hoc* test showed significant group difference between aMCI *v.s.* AD. *Post hoc* pairwise comparison was performed through Fisher’s least significant difference (LSD) and *p*<0.05 was considered significant. **Bold** data indicated statistical significance.

### 2.2 Small-world Network Metrics

**Figure 2:**
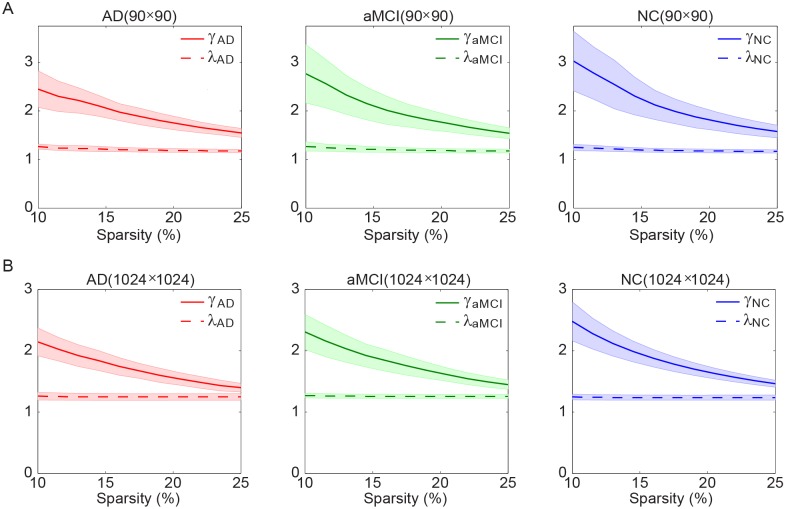
Small-world network metrics of functional brain networks at two different parcellation scales. The graphs show the changes in *γ* (solid line) and *λ* (dashed line) in the functional brain networks of AD (red), aMCI (green), and NC (blue) groups at (A) low parcellation scale and (B) high parcellation scale with respect to sparsity. For all the three groups, *γ* was significantly greater than 1 and *λ* was relatively close to 1, indicating prominent small-world properties for all groups.

Previous studies have demonstrated that small-world topology exists in both functional brain networks [8], [34] and structural brain networks [9], [35] of human and nonhuman primates [36]. The small-world properties of three groups (AD, aMCI, & NC) are shown in Figure 2. Over a wide range of sparsity (10%–25%), all three groups exhibited small-world properties as they had a larger clustering coefficient (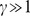) and an almost identical shortest path length (*λ*≈1) compared to the size-matched random networks. This small-world property suggests that in all three groups the brain networks are efficient for both global and local information transfer. In agreement with previous studies [8], [9], [34], [37], [38], the functional networks of the patients with AD and aMCI showed varying degrees of degeneration of this efficient architecture. Statistical results of the global network metrics at a specific sparsity (*S* = 10%) were summarized in Table 2. Significant group effect was revealed in the normalized clustering coefficient (*γ*), and small-worldness (*σ*) regardless of the parcellation. *Post hoc* comparisons showed significantly reduced *γ* and *σ* in AD patients relative to the controls while such corresponding metrics were intermediate for the aMCI group. More interestingly, only in the high resolution parcellation, a significant (*p*<0.05) decrement of *σ* was revealed in aMCI group compared to healthy subjects. In addition, significant positive correlations were identified between*γ* and MMSE as well as *σ* and MMSE at two parcellation scales. Our findings thus provided further support for the hypothesis that brain network had a decline in the optimal small-world architecture in the progression from MCI to AD [8], [9]. AD-related differences in global network metrics were not unique to the single sparsity used to generate low-cost networks with *S* = 10%. As shown in Figure 2, AD patients had reduced *γ* and *σ* over almost the entire range of the network sparsity (10%–25%). The integrals of the network metrics (corresponding to the areas of the network metrics curves within the sparsity range from 10%–25%) were used as summary metrics and confirmed detrimental effects of AD on clustering coefficients and small-worldness independent of different parcellation scales (Table S2).

**Table 2:** Comparisons of the global network measures among the AD, aMCI and NC groups at sparsity of 10%.

				ANOVA		Correlation with MMSE	
	AD	aMCI	NC	F value	*p* value	*r* value	*p* value
Low parcellation (90-ROIs)							
*γ*	2.45 (0.38)	2.77 (0.40)	3.03 (0.41)	3.43	**0.04** †	0.36	**0.02**
*λ*	1.27 (0.05)	1.27 (0.10)	1.25 (0.06)	0.41	0.67	−0.11	0.48
*σ*	1.94 (0.34)	2.19 (0.51)	2.43 (0.47)	3.72	**0.03** †	0.37	**0.02**
High parcellation (1024-ROIs)							
*γ*	2.15 (0.23)	2.31 (0.29)	2.48 (0.32)	4.62	**0.02** †	0.46	**<0.01**
*λ*	1.26 (0.06)	1.27 (0.04)	1.25 (0.05)	0.87	0.43	0.05	0.77
*σ*	1.70 (0.20)	1.82 (0.25)	1.99 (0.26)	5.29	**0.01** * **^,^** †	0.41	**0.01**

Values are represented as the mean (S.D.). For each participant, two functional connectivity networks were obtained via different parcellation scales (low: 90×90, and high: 1024×1024). The comparisons of the integrated global network measures among the three groups (AD, aMCI, & NC) were estimated using ANOVA. The bivariate correlation of the network metrics with MMSE was performed using Pearson’s correlation.

*Post hoc test showed significant group difference between control *v.s.* aMCI;

† *Post hoc* test showed significant group difference between control *v.s.* AD;

*Post hoc* pairwise comparison was performed through Fisher’s least significant difference (LSD) and *p*<0.05 was considered significant. **Bold** data indicates statistical significance.

### 2.3 Altered Regional Nodal Characteristics in aMCI and AD

**Figure 3:**
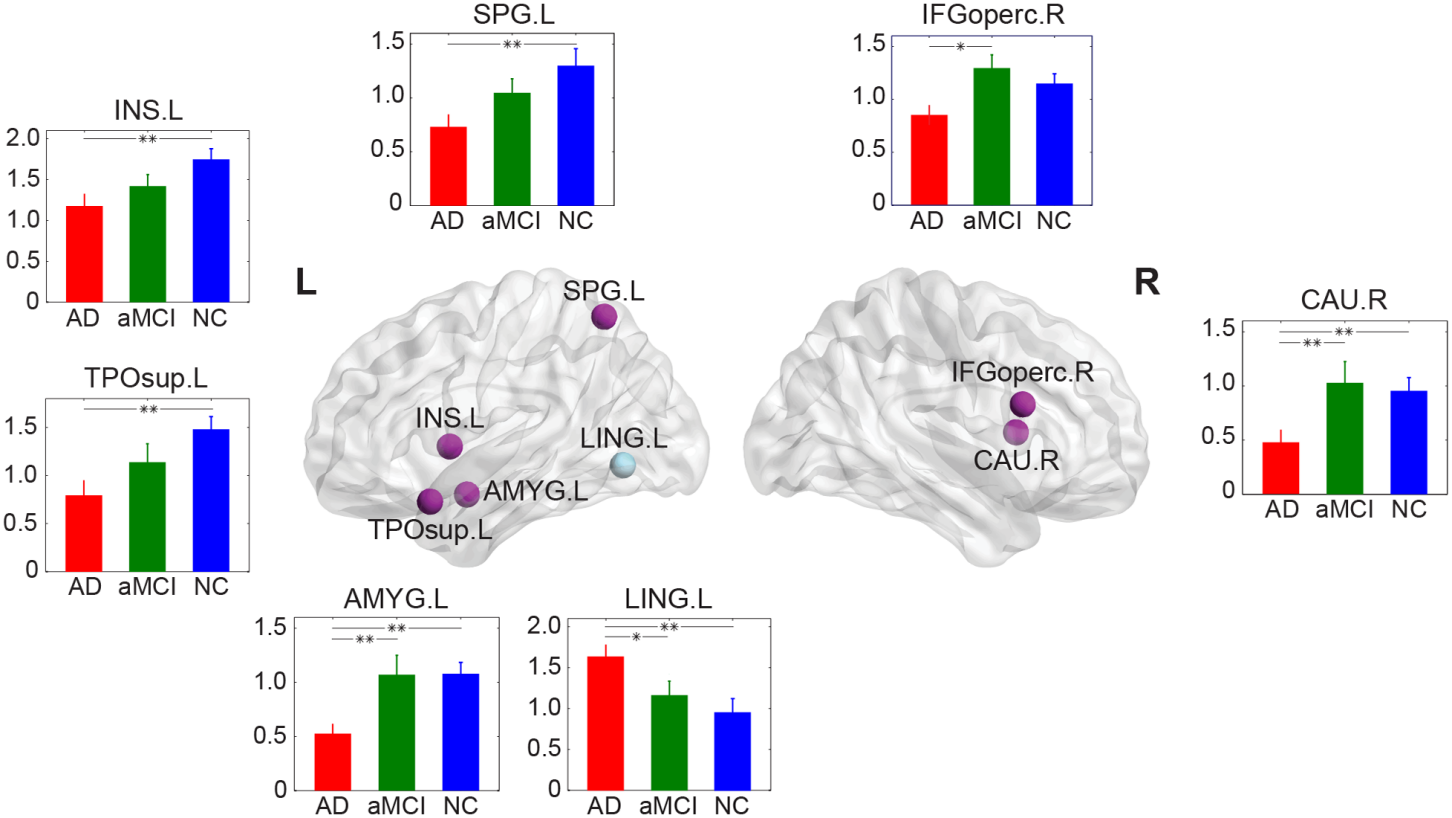
The distribution of brain regions with both significant (*p*<0.05, uncorrected) groups effects in BC and significant (*p*<0.05) correlation between BC and MMSE scores. Each bar represents the average BC values for AD (red), aMCI (green), and NC (blue). Error bars represent standard deviations (S.D.). The regions were overlaid on the brain surface at the Medium view. *Post hoc* pairwise comparison was performed through Fisher’s least significant difference (LSD) with * indicates *p*<0.05 and ** indicates *p*<0.01. Regions showing positive correlation are coded in purple and region showing negative correlation is coded in light grey. For the abbreviations of cortical regions, see Table S1. L = left; R = right. The figure was visualized with BrainNet Viewer software (http://www.nitrc.org/projects/bnv/).

Following the discovery of a less efficient global network organization in patients with aMCI and AD, we further investigated the localized alteration (i.e., nodal efficiency) of the network structure. Regions with significant group effects and correlation (Pearson’s *p*<0.05) with clinical diagnostic result (MMSE) are shown in Figure 3, including two regions in the right hemisphere (opercular part of inferior frontal gyrus (IFGoperc.R) and caudate nucleus (CAU.R)) and five left hemispheric regions [superior parietal gyrus (SPG.L), insula (INS.L), temporal pole, superior temporal gyrus (TPOsup.L), amygdala (AMYG.L), and lingual gyrus (LING.L)]. Additionally, post hoc tests unfolded that most of them (5 of 7: SPG.L, INS.L, TPOsup.L, AMYG.L, and CAU.R) had a significant nodal efficiency decreases in AD patients compared to health controls. Group differences between AD and aMCI were uncovered in three regions (IFGoperc.R, CAU.R and AMYG.L), with a higher nodal efficiency observed in the aMCI group. More interestingly, one region (LING.L) showed a significant increased nodal efficiency in AD patients compared to aMCI and NC subjects. Together, our results are consistent with several recent studies showing that the roles of regions in managing information flows over the brain networks were profoundly affected in AD and aMCI patients [8], [9], [37].

### 2.4 Modularity and Disease-related Changes

**Figure 4:**
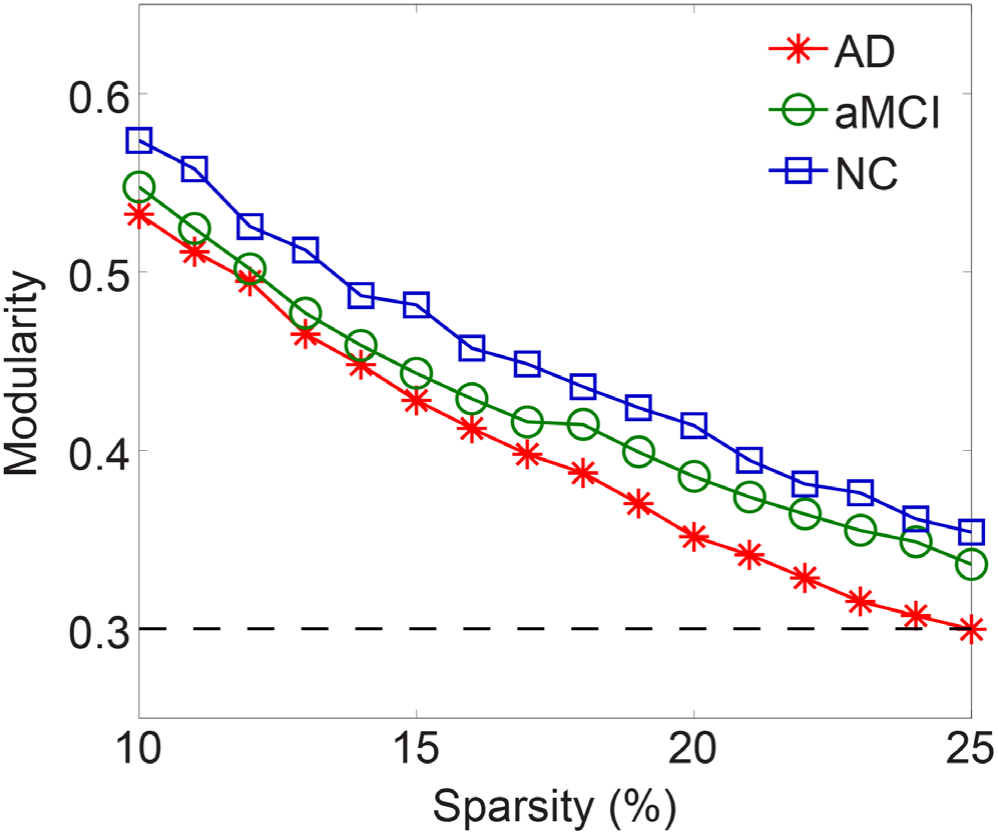
Modularity Q of the functional brain networks for AD, aMCI and NC groups with respect to sparsity. The black dashed line indicates the threshold for the nonrandom community structure. Note that only the modularity of AD group is less than 0.3 for *S* = 25%.

**Figure 5:**
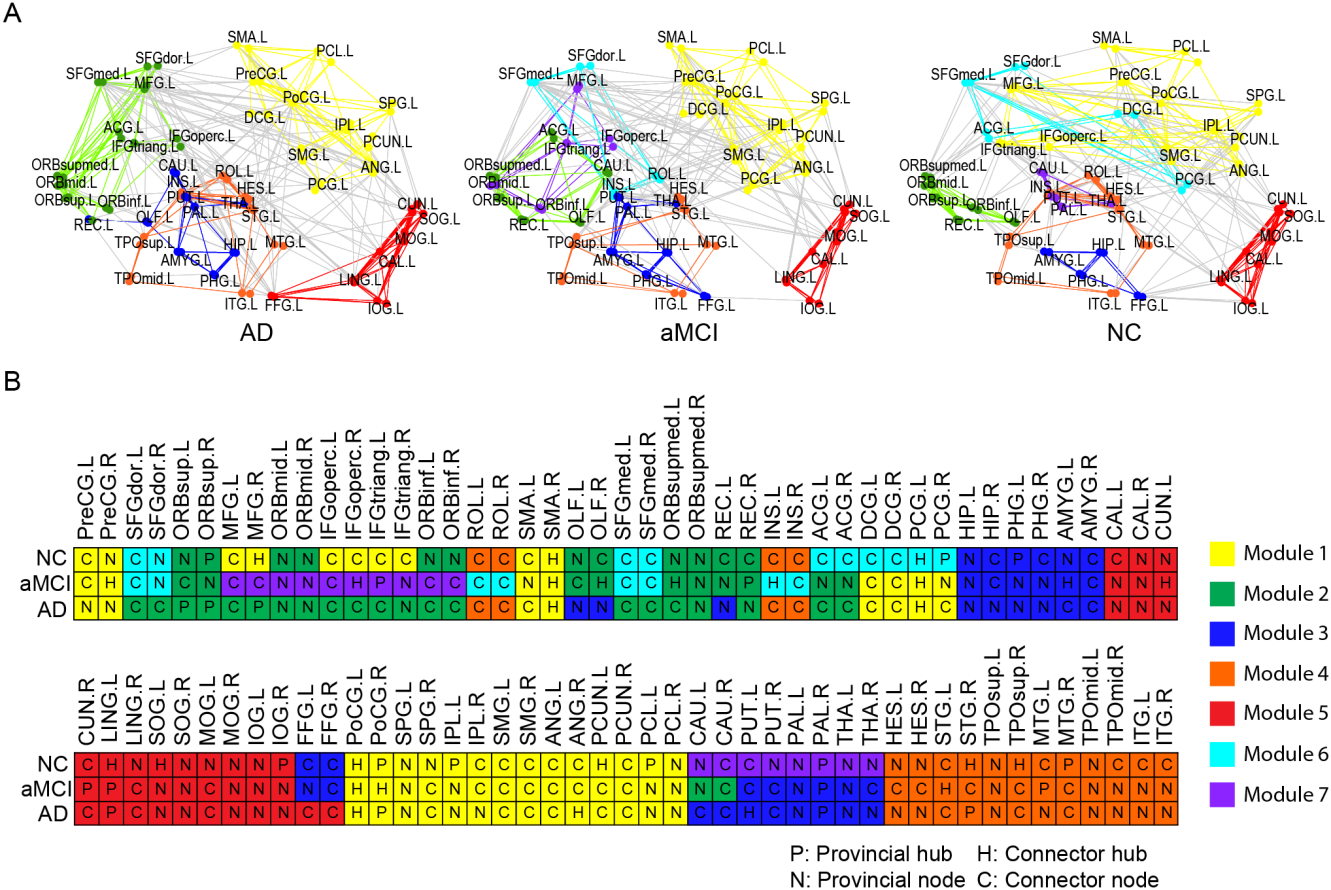
The modular structure of group averaged functional brain network at *S* = 10%. (A) Color coded modular structure plotted in the anatomical space. The brain network is presented with the frontal cortex on the left of the panel and the occipital region on the right of the panel. Inter-modular links are colored in gray for better illustration. Detailed information about the module regions and regional node roles for AD, aMCI and NC groups are presented in (B). The module order was determined according the descending order of the region numbers in AD group. The topological representations were drawn by the Pajek (http://pajek.imfm.si/doku.php). For the abbreviations of cortical regions, see Table S1. L = left; R = right.

Maximum modularity *Q* of the brain networks decreased as a function of increasing sparsity (Figure 4). In a wide sparsity range, a decline trend with the cognitive impairment was observed (*Q_AD_* <*Q_aMCI_* <*Q_NC_*). A community structure would be considered as nonrandom community if its modularity fulfills: *Q*≥0.3 [39]. In the current work, the functional brain networks in the three groups were consistently modularly organized (*Q*≥0.3 over the predefined sparsity band). The community structures were obtained for the three groups at a fixed sparsity (*S* = 10%), which captured the connectivity backbone and maintained a fully-connected brain networks. The brain networks were separated into five, seven, and seven modules for AD, aMCI and NC groups, respectively (see Figure 5A for their color coded modular structures in the anatomical space). According to their topological functions in the network, four possible roles of the regions were defined as connector hub (H), provincial hub (P), connector node (C), and provincial node (N). The information about the module compositions and regional node roles for each group was presented in Figure 5B. The distribution of the connectors and the topological roles of the module of three groups were summarized in Table 3.

**Table 3:** The distribution of connectors and the topological roles of the modules among the AD, aMCI and NC groups.

	AD			aMCI			NC		
Module	Regions	Connectors	Intermodule	Regions	Connectors	Intermodule	Regions	Connectors	Intermodule
1	22	**14 (0.64)**	**65 (0.27)**	22	**16 (0.73)**	**78 (0.27)**	24	**17 (0.71)**	**76 (0.29)**
2	21	**13 (0.62)**	**68 (0.28)**	12	5 (0.42)	26 (0.09)	12	3 (0.25)	20 (0.08)
3	17	6 (0.35)	26 (0.11)	14	7 (0.50)	24 (0.08)	8	5 (0.63)	15 (0.06)
4	16	7 (0.44)	41 (0.17)	12	6 (0.50)	37 (0.13)	16	**11 (0.69)**	**41 (0.16)**
5	14	5 (0.36)	42 (0.17)	12	3 (0.25)	27 (0.09)	12	4 (0.33)	29 (0.11)
6				8	**7 (0.88)**	**73 (0.25)**	10	**8 (0.80)**	**60 (0.23)**
7				10	6 (0.60)	25 (0.09)	8	2 (0.25)	23 (0.09)
Total	90	45	121	90	50	145	90	50	132

Values in the “Regions” column are the region number within each module. The “Connector” column represents the numbers of connector nodes (C and H) in each module and its ratio regarding to the total number of regions in such module. The values in the “Intermodule” column indicate the numbers of intermodule edges and its ratio to the total number of intermodule connections in all modules. Bold date indicates the module with both the higher connector coefficient (>0.6) and the higher intermodule connections (>1/number of modules) [19].

#### 2.4.1 Modules and node roles in functional brain network with AD

The functional brain network of AD patients comprised 5 connected modules, which varied in size from 22 to 14 regional nodes (Table 3). For Module 1, most of the regions (14 among 22) had numerous connections to other modules. Four of them were categorized as connector hubs (SMA.R, PCG.L, PoCG.L, and ANG.R) because they also had high intra-module connections. Only 8 among 22 regions had no connection to regions in other modules, including one provincial hub (PoCG.R). Similar to Module 1, most regions (13 among 21) in Module 2 were identified as connectors, including one connector hub (MFG.L); the other 8 among 21 regions were categorized as provincial nodes, including 3 provincial hubs (ORBsup.L, ORBsup.R, and MFG.R) with high intra-modular connections. While, for Module 3, most (11 among 17) regions were provincial nodes, including 1 provincial hub (PAL.R); only 6 regions were categorized as connectors, including 1 connector hub (PUT.L). For Module 4, most regions (9 among 16) were identified as provincial nodes, including 1 provincial hub (STG.R). Likewise, for Module 5, only 5 among 14 regions were categorized as connector nodes.

#### 2.4.2 Modules and node roles in functional brain network with aMCI

The functional brain network of aMCI comprised 7 modules, varying in size from 22 to 8 regions (Table 3). Most of the regions (16 among 22) in Module 1 were identified as the connectors. Five regions (PreCG.R, SMA.R, PCG.L, PoCG.L, and PoCG.R) were categorized as connector hubs. Only 6 among 22 regions had no inter-module connection and were categorized as provincial nodes. For Module 2 in aMCI patients, most regions (7 among 12) were categorized as provincial nodes, including 1 provincial hub (REC.R); the other 5 regions were categorized as connectors, including 2 connector hubs (OLF.R and ORBsupmed.L). For Module 3, half of the regions (7 among 14) were classified as connectors, including 1 connector hub (AMYG.L). The rest 7 regions only have local connections, and were categorized as provincial nodes, including 1 provincial hub (PAL.R). Likewise, for Module 4, half of the regions (6 among 12) were categorized as connectors, with 1 connector hub (STF.L). Among the rest 6 provincial nodes, MTG.L was classified as provincial hub. Only 3 among 12 regions were categorized as connectors in Module 5, including 1 connector hub (CUN.L); most the regions (9 among 12) were identified as provincial nodes, including two provincial hubs (CUN.R and LING.L). Interestingly, most of the regions were categorized as connectors in Module 6 (7 among 8) and Module 7 (7 among 10).

#### 2.4.3 Modules and node roles in functional brain network of NC

The group averaged brain network of NC subjects also comprised 7 modules, varying in size from 24 to 8 regions (Table 3). For regions comprising the Module 1, most (17 among 24) regions were categorized as connector nodes, including four connector hubs (MFG.R, SMA.R, PoCG.L, and PCUN.L); only 7 among 24 regions had no inter-module connection and were classified as provincial nodes, including three provincial hubs (PoCG.R, IPL.L, and PCL.L). For Module 2, only 3 among 12 regions were categorized as connector nodes, with no connector hubs; most (9 among 12) regions were identified as provincial nodes, including 1 provincial hub (ORBsup.R). For Module 3, most (5 among 8) regions were categorized as connectors, without connector hubs; the rest (3 among 8) regions were classified as provincial nodes, with 1 provincial hub (PHG.L). Module 4 included exactly the same set of brain regions as in AD group. However, the topological role profile of the module is significantly different, that is, most (11 among 16) regions were categorized as connectors, including 2 connector hubs (STG.R and TPOsup.R); only 5 among 16 regions were classified as provincial nodes, including 1 provincial hub (MTG.R). For module 5, only 4 among 12 regions had inter-module connections, including 2 connector hubs (LING.L and SOG.L); most (8 among 12) regions were categorized as provincial nodes, including 1 provincial hub (IOG.R). Most (8 among 10) regions in Module 6 were classified as connectors, including 1 connector hub (PCG.L); the rest 2 regions included 1 provincial hub (PCG.R). The regions in Module 7 are all subcortical regions, where only 2 among 8 regions were connectors, with no connector hub; the rest 6 regions only have local connections, including 1 provincial hub (PAL.R).

By inspection of Figure 5, all three groups showed clear similarities in relative size and composition of the modules, suggesting that certain modular organization is conserved in AD. For instance, Module 1 in all groups comprised almost same brain regions and most of the regions in this module served as connector-module (Table 3). Such similarities were also found in the Module 4 and Module 5. Nevertheless, there were also significant changes in the topological roles of regions and modules. Module 2 in AD group were re-organized and segregated into two modules (i.e., Module 6 and Module 7) in aMCI group. Therefore Module 2 in aMCI group included a smaller number of regions; the topological role of “connector-module” was inherited by Module 6 in aMCI group (See methods section of the definition of connector-module). In addition, the regions constituting Module 7 of the NC brain network were a subgroup of the brain regions constituting Module 3 in AD and aMCI and were all subcortical brain regions (bilateral caudate nucleus, lenticular nucleus (both putamen and pallidium parts), and thalamus). Note that Module 7 in aMCI and NC groups had no overlap brain regions. Interestingly, the topological role of Module 4 in NC group was different (served as connector-module) compared to AD and aMCI group, though it included almost the same set of brain regions. Moreover, significant differences in the topological roles of the modules in the brain networks of three groups were also revealed. The connector-module was defined as the module that had both high connector ratio and a high ratio of intermodule connections. For patient groups, only two modules (Module 1 & 2 for AD, Module 1 & 6 for aMCI) were identified as connector-module. The NC group showed a higher proportion of intermodule connections and more connectors, e.g., three modules (i.e., Module 1, 4, & 6) were classified as connector-module.

### 2.5 Functional-structural Connectivity Relationship

**Figure 6:**
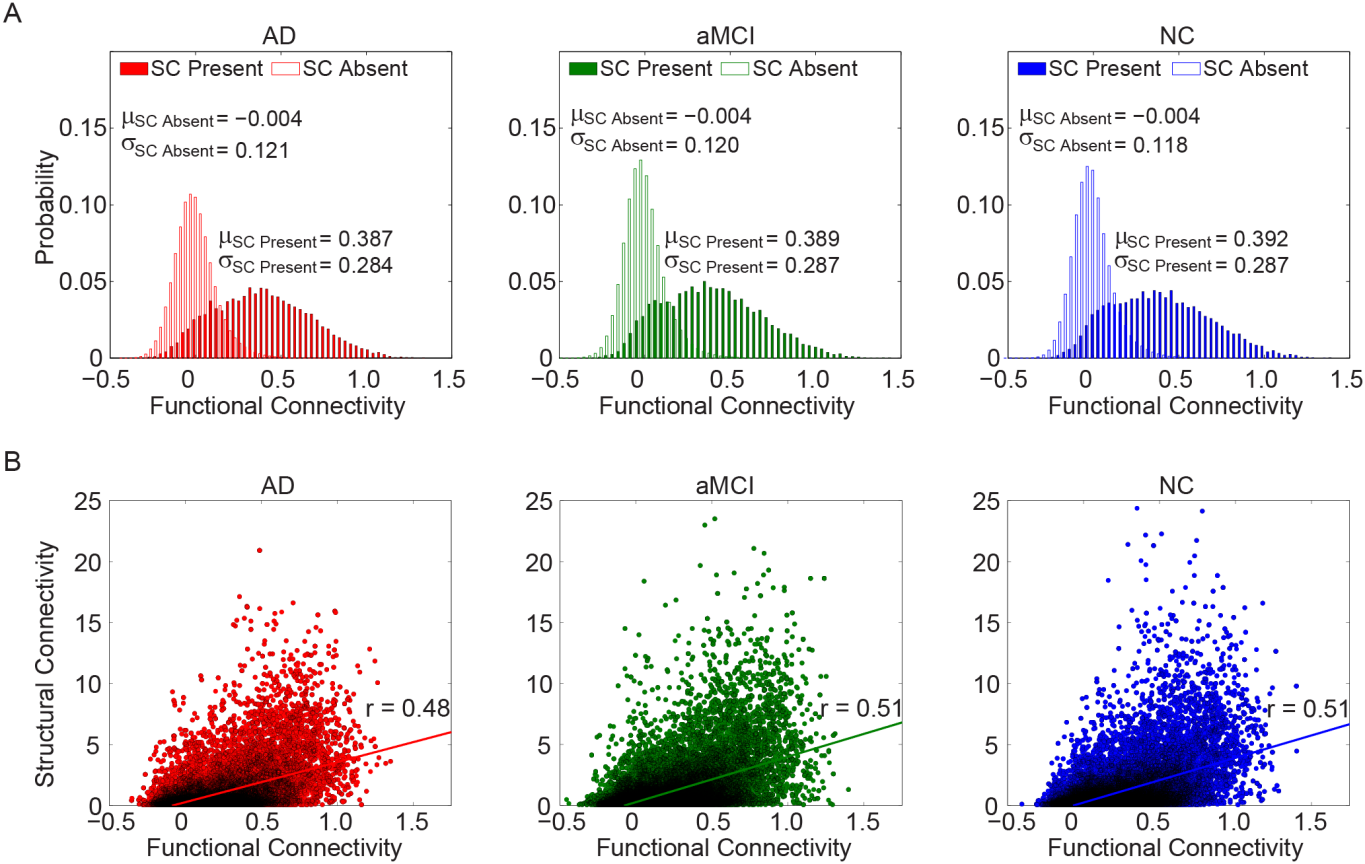
The overall FC-SC relationships. (A) The probability densities of FC strengths between structurally connected and unconnected regions pairs. When direct SC presents, higher FC strength appears than the case direct SC absents, indicating a robust relationship between FC and SC. (B) Scatter plot of SC strength against FC strength for the regions with direct SC. Further analysis showed significant coefficient between FC and SC strengths for all groups. Data are from group averaged functional connectivity matrix of AD, aMCI, and NC groups, at the high resolution (1024×1024).

After averaging connectivity data across all subjects in the same group, the probability densities of FC were categorized into two classes: with SC and without SC. As shown in Figure 6A, when direct SCs are present, the strength of FC is evidently larger than 0 (i.e., mean value (µ) larger than 0) for all three groups; while when direct SCs are absent, the strength of FC varies over a wide range around 0 (i.e., with a mean value close to 0). Those findings are consistent with observations demonstrated in [26], [40]. Interestingly, when direct SC is present, µ values of the FC distribution presents an increase trend among three groups (AD<aMCI<NC). However, for individual participants, the group effect of µ values failed to pass the significant level.

**Figure 7:**
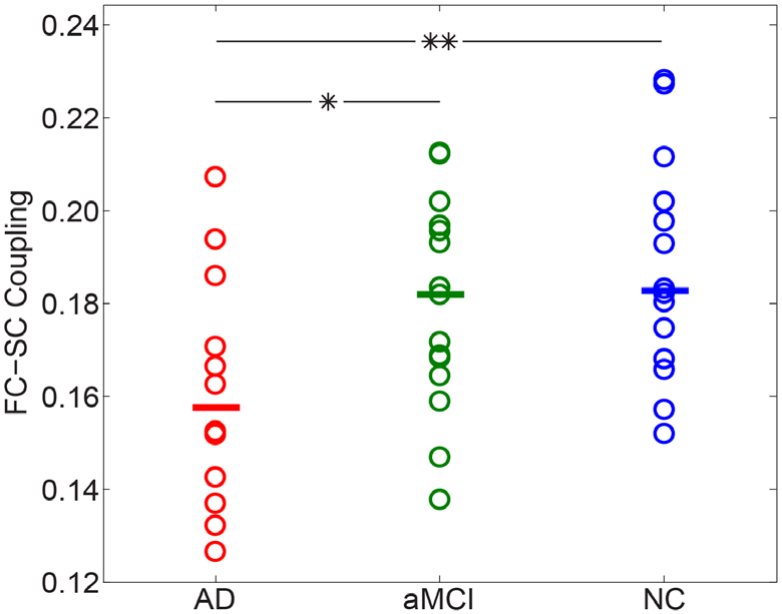
The functional connectivity (FC) – structural connectivity (SC) coupling for AD patients, aMCI patients, and healthy controls. AD patients exhibited a significant decrease in FC-SC coupling compared to aMCI patients and healthy controls. Significant statistical differences are indicated with * for *p*<0.05 and ** for *p*<0.01.

For data averaged across participants in each group, the FC-SC correlation was *r* = 0.35 for AD, *r* = 0.36 for aMCI, and *r* = 0.37 for NC (Figure not shown). When excluding region-pairs without SCs, the correlation of group averaged data strengthens to *r*>0.48 for all groups (see Figure 6B). For individual participants, the FC-SC correlation coefficient ranged from 0.13 to 0.23 (Figure 7). The detrimental effect of AD on the relationship between FC and SC strength was statistically significant (ANOVA, F = 4.17, *p* = 0.02). *Post hoc* comparisons showed significantly (*p*<0.05) reduced FC-SC relationship in AD group relative to the aMCI patients and controls.

## Discussion

In this study, we investigated the changes of functional brain networks associated with AD by graph theoretical analysis, and found evidence that mostly supported our hypotheses. In particular, three main findings were found. First, the organization of the functional brain networks in aMCI and AD patients were significantly disrupted, as indicated by reduced clustering coefficient and small-worldness. Moreover, the characteristics of brain network in the aMCI patients displayed an intermediate position between those of NC and AD patients. In addition, the results on functional impairment of regional characteristics suggested that AD related cognitive impairments might be related to the less efficient information transfer. Second, we demonstrated that functional brain networks exhibited modular organization in all groups and changed greatly in the AD group. Compare to NC, who represented a distributed organization, brain networks of AD patients showed a more localized modular organization. Third, we also reported, for the first time, a decline in the FC-SC coupling in the progression from MCI to AD. Taken together, these results extend our understanding of the neurophysiologic mechanisms associated with aMCI and AD from a network perspective.

### 3.1 Small-world Properties and AD-related Changes

Our results showed that the resting-state brain networks of aMCI and AD patients also exhibited a small-world topology, which agrees well with previous fMRI studies on AD [8], [11], [34]. Note that though functional brain network of aMCI and AD patients showed prominent small-world properties like that of NC, the normalized clustering coefficient (γ) and small-worldness (σ) were significantly lower in aMCI and AD compared to NC in both low and high parcellation scales. Clustering coefficient represents efficiency of local information transfer for specialized processing, which is believed to constitute the basis of cognitive processes. The disease related decrease in the normalized clustering coefficient and small-worldness could be attributed to the hypo-metabolism in aMCI and AD [41]. We also observed a progression pattern of the altered network metrics from aMCI to AD, i.e., γ and σ of aMCI patients showed median values between those of NC and AD patients. The lower clustering and small-worldness in the aMCI and AD networks indicate that normal balance between local specialization and global integration was disturbed. Our result thereby further supports the notion of AD as a disconnection syndrome from a network perspective. More importantly, we revealed that aMCI forms a boundary between normal aging and AD and represents a functional continuum between healthy aging and the earliest signs of dementia.

### 3.2 Disease Related Distinctions of Nodal Characteristics

Following the discovery of a disrupted global network organization in aMCI and AD, we further localized the brain regions exhibiting significant altered nodal betweenness which also correlates with the neuropsychological MMSE scores. Among the three groups, we found that the regions with significant group effects were mainly association and paralimbic cortex regions (Figure 3). Most (6/7) regions showed significantly decreased nodal centrality in AD; among them four brain regions (SPG.L, INS.L, TPOsup.L, and AMYG.L) exhibited monotonically reduced nodal betweenness with the progression of AD. Previous studies have found that subjects with aMCI and AD had a significant loss in the amount of gray matter in hippocampus, caudate nucleus, insula, medial temporal lobe, parietal areas, and frontal cortex [2], [42]. Therefore, the reduced nodal betweenness centrality might be attributed to the regional gray matter reduction. Interestingly, one region (LING.L) showed significantly increased nodal centrality in AD. There is evidence for AD-related increases in blood-oxygen-level dependent (BOLD) signal [43] and functional connectivity [33] of lingual gyrus during cognitive tasks. In addition, He and colleagues had also revealed an increase of nodal centrality in lingual gyrus in structural brain networks in AD [9]. We speculated that the increased betweenness centrality of LING.L in AD might represent a compensatory process for the reduced centrality in the regions described earlier. Together, our results suggested that the roles of brain regions in managing information transmission and functional integration over the networks were profoundly affected in AD patients.

### 3.3 Abnormal Changes in Modularity

Resting-state functional brain networks have been described consistently, indicating the existence of a modular organization [13], [44], [45]. The highly modularized architecture elucidated in the current study hence provided further support for the presence of the modular structure in the functional brain network of healthy controls, aMCI and AD patients. It has been suggested that such modular organization contribute to various aspects of intrinsically functional organization of human brain such as the balance between brain functional segregation and integration while conserving wiring cost and high resilience to network node or edge damages [4]. The number of modules, five to seven modules, we observed in our networks resembled what was found in previous fMRI studies [13], [44], [45]. Key circuit components related to the primary brain functions such as motor, auditory and visual systems, were consistently detected in previous studies [13], [19]–[21], [45]. Our results are also consistent with these findings: Module 1 was primarily associated with motor and somatosensory; Module 3 was mainly involved in the visual system; Module 4 was involved in auditory and memory functions in all three groups. Despite these convergence results, there were notable discrepancies in the composition and topological roles of modules among the brain networks in this study. The composition of the modules was quite different among three groups (Figure 5). Two new modules (Module 6 & 7) were identified in aMCI group, representing the re-organization of the brain regions known as Module 2 and Module 4 in the AD group. In NC group, two new modules (Module 6 & 7) were revealed, representing the reorganization and separation of subcortical regions from the areas known as Module 2 and 3 in AD group. In other words, the regions in the AD group were assembled more densely, leading to the overnumbered regions in modules. In addition, the modules in the aMCI and AD groups seemed to be more locally organized, resulting in fewer connectors and inter-module connections. The connectors were crucial for maintaining network integrity; and intermodule connections facilitated communication between different modules [19], [45]. Three connector-modules were identified in NC, while only two were revealed in aMCI and AD groups. These findings pointed in the direction similar to the results of age-related changes in modular organization of human functional brain networks [19], [20], which indicated the organization of multiple functional networks shifts from distributed organization in middle-age groups to a more localized organization with great alternation in old age.

### 3.4 Altered Functional-structural Relationship in aMCI and AD

We observed an evident relationship between the strengths of FC and SC in all three groups when structural connections are present. This finding agrees with previous studies and provides further support that functional linked resting-state connectivity is reflective of the underlying structural connectivity architecture [26], [46]. We further assessed the possibility of inferring the presence of SC through thresholding FC and found that the prediction was not reliable (data not shown), indicating that the functional and structural organization of the brain network is not a one-to-one relationship [4], [26], [47]: the anatomically unconnected edges also exhibit FC with strength in a wide range (Figure 6A). The presence of FC in the absence of SC may reflect that two functional connected cortical regions may be connected via a third region without a direct structural connection.

More interestingly, a decreased coupling between FC and SC was observed in AD patients. Further analysis reveals that the overall level of SC strengths was significantly reduced in AD patient groups (data not shown), supporting previous observations [7]; yet the FC strengths did not show significant group effects. We speculated that the decreased correlation between FC and SC in AD may attribute to (i) the asynchronous decrease of the FC and SC, and (ii) functional interactions are less directly related to the underlying anatomical connections in AD. The decreased FC-SC coupling may be indicative of more stringent and less dynamic brain function in AD patients. It is notable that this is the first work, to our knowledge, which finds a decreased relationship between FC and SC in AD. We demonstrate that AD related brain dysfunctions can be characterized by profiles of FC – SC relationship.

### 3.5 Methodological Issues

There are several issues that should be addressed. First, it has been suggested that functional brain networks share similar topological features with anatomical networks, implying a close relationship between brain function and structure. Although the results of the current study mainly obtained from functional brain networks, similar findings have been revealed in the structural brain networks, further proving that AD is a disconnection syndrome (R. Fang, et al., unpublished data). Combining different modalities would aid in uncovering how the functional brain network changes are associated with underlying anatomical changes in aMCI and AD. Second, recent studies have suggested that the node definition by different parcellation scales might result in different properties of brain networks [48]–[52]. In the current study, two parcellation scales were adopted and the network analysis of global properties corresponding to these two scales showed comparable results, i.e., in both parcellation scales, small-world network properties were decreased with AD progress. Graph theoretical analysis with different spatial resolution would be necessary and important to provide more comprehensive information of the topological alterations of the brain networks in AD patients. Note that the comparison of network parameters across studies must be made with reference to the spatial scale of the nodal parcellation [48]. Third, we constructed weighted functional brain networks with estimating the Pearson’s correlation coefficients between the time series of BOLD signals in the frequency interval of 0.01–0.08 Hz between all possible pairs of regions. One recent study reported that certain subset of frequency band, i.e., 0.031–0.063 Hz, is more sensitive for detecting the topological aberrations in aMCI patients [53]. Accumulating evidence also revealed frequency-dependent functional changes in the brain under various clinical conditions [54], [55]. Therefore, future studies considering the frequency specificity will provide a comprehensive understanding about the pathophysiological mechanisms of aMCI and AD. Finally, considering the small number of subjects was used in this study, a further study is needed to assess the reproducibility of the obtained results with a large number of subjects.

To summarize, we quantitatively analyzed the changes in small-world properties and modularity of functional brain networks in NCs, aMCI and AD patients. Our results of global network metrics indicated that although the overall small-world property is preserved in aMCI and AD patients, clustering coefficient and small-worldness are progressively decreased during AD development. We also demonstrated that inter-modular connections seem to be vulnerable in aMCI and AD, suggesting the relevance of a network perspective on dementia and further proving AD as a disconnection disease. More importantly, we found, for the first time, a dissociated relationship between functional and structural connectivity in AD. We interpret our finding as a proof of principle, providing insightful implications on how the brain regions interact abnormally in patients with aMCI and AD, and demonstrating that functional brain disorders can be characterized by multimodal neuroimaging-based metrics.

## Materials and Methods

### 4.1 Participants

Participants for this study were 41 right-handed subjects, comprising 12 patients with AD (4 males), 15 patients with aMCI (7 males) and 14 sex-, age-, and education-matched normal control subjects (NC, 6 males) recruited from the neurology department of Ruijin Hospital, Shanghai Jiao Tong University, Shanghai, China. All participants were native Chinese speakers. Written informed consent was obtained from each participant following a complete description of the study, and the study was approved by the Institutional Review Board of the Ruijin Hospital. Ethics review criteria conformed to the Declaration of Helsinki. Diagnostic evaluation was performed by a board-certified neurologist using information obtained from the clinical history, mental statues examination, existing medical records, and the administration of the DSM-IV disorders-Patients Version (SCID-P). All participants were assessed using a standardized clinical evaluation protocol that included the Mini-Mental State Examination (MMSE) [56], the Clinical Dementia Rating Scale (CDR) [57], the Activities of Daily Living Scale (ADL) [58], and the Addenbrooke’s Cognitive Examination Revised (ACER) [59]. The diagnosis criteria of each group were the following: (1) AD group: participants should meet the McKhann criteria of probable AD of the National Institute of Neurological and Communicative Disorders and Stroke and Alzheimer Disease and Related Disorders Association (NINCDS-ADRDA) [60]; (2) aMCI group: subjects should meet the Petersen’s criteria [1] and exhibited objective evidence of impaired memory compared to normal subjects matched for age, gender and education (the Auditory Verbal Learning Test (AVLT of Chinese version) [61], AVLT-delayed scores: ≤4, between 51–60 years; ≤3, between 61–70 years; and ≤2, over 71 years, see Table S3); (3) NC group: control subjects denied any significant neuropsychiatric disease or memory problem and were not taking any psychoactive medication. Healthy subjects with a MMSE score less than 26 and patients with neurologic disease other than AD and aMCI, which might impair the current study, were not eligible for the study. All magnetic resonance imaging data were collected immediately upon diagnosis. The detailed demographics and clinical characteristics of the participants were presented in Table 1.

### 4.2 Data Acquisition

MRI data were acquired using a GE 3-Tesla MR scanner (Signa HDxt 3T, GEMS, GE Healthcare) in the radiology department of Ruijin Hospital, Shanghai Jiao Tong University, Shanghai, China. Functional data was obtained using a single-shot echo-planar imaging (EPI) sequence and the acquisition parameters consisted of the following: repetition time [TR] = 3000 ms, echo time [TE] = 40 ms, field of view [FOV] = 24×24/Z, flip angle = 90°, matrix size = 64×64, NEX = 1. Twenty-seven slices (5 mm slice thickness with no gap in-between) were acquired in an inferior to superior direction in sequential order. During the data acquisition, participants were required to lie quietly in the scanner with their eyes closed to minimize motion artifacts. The scan included 120 volumes for each participant. For each subject, three dimensional high resolution T1-weighted images were also obtained by a magnetization prepared rapid acquisition gradient echo (MPRAGE) sequence with the following parameters: TR = 5.6 ms, TE = 1.8 ms, flip angle = 15°, matrix size = 256×256, voxel resolution = 1×1×1 mm^3^.

### 4.3 Data Preprocessing and Network Construction

Data preprocessing was carried out using Statistical Parametric Mapping (SPM8, http://www.fil.lon.ucl.ac.uk/software/SPM8/; Wellcome Trust Center for Neuroimaging, University College London), resting-state fMRI data analysis toolkit (REST) [62] and Data Processing Assistant for Resting-State fMRI (DPARSF) [63] running under Matlab 2011a. Briefly, prior to preprocessing, the first 10 volumes were discarded considering the instability of the initial signals and the subjects’ adaption to the environment. The remaining fMRI volumes were corrected for different signal acquisition times by shifting the signal measured in each slice relative to the acquisition of the slice at the mid-point of each TR. Then, the time series of images for each subject were realigned to the first volume (i.e., the original 11^th^ volume) to compensate the inter-scan head motion artifacts using a least squares approach and a six-parameter linear transformation [64]. Exclusion threshold for excessive head movement was set as>3 mm or 3° in this study. All subjects were eligible for these criteria. The relative displacements (FD-Jenkinson) for all three groups were found to be small (mean ± S.D. for AD: 0.079±0.056 mm, aMCI: 0.071±0.032 mm, and NC: 0.078±0.043 mm). No statistical differences were found among three groups (ANOVA, F = 0.161, *p* = 0.852). A standard template (Montreal Neurological Institute) was then employed to normalize the resulting motion-corrected functional volumes [65], which were further resampled to 3×3×3 mm^3^ and spatially smoothed by convolution with an isotropic Gaussian kernel (FWHM = 4 mm). The temporal waveform of each voxel was finally band passed into 0.01–0.08 Hz to reduce the effect of every low frequency drift and high frequency physiological noise. For more details about the data preprocessing, please refer to [63].

To study the topological properties of functional brain networks among three groups, we examined the correlation matrices using graph theoretical analysis. In the current study, two parcellation of regions of interest (ROIs) (low: 90 ROIs, and high: 1024 ROIs) were employed to investigate the dependence of functional brain networks on different nodal scales. To construct the brain networks on low resolution, we employed the automated anatomical labeling (AAL) atlas to parcellate the brain into 90 ROIs (denoted as AAL-90, 45 for each hemisphere, see Table S1) [66]. Prior to the BOLD signal estimation, the whole-brain signal, cerebrospinal fluid and white matter signals were removed as nuisance variables to reduce the effect of the physiological artifacts and non-neuronal BOLD fluctuations [67]. Representative time series (110 time-points) of each ROI was then obtained by averaging the time series of each voxel with that region. Pearson correlation coefficient, which represented the functional connectivity strength, was then calculated between all possible pairs of ROIs. A Fisher’s-Z transformation was applied to the correlation matrices to improve the normality of the correlation coefficients [68] and further reduce the relationships between motion and inter-individual differences in the obtained functional connectivity matrixes [69]. For high resolution ROI partition, a template of 1024 ROIs (called AAL-1024), which are with almost equal size of brain regions and sub-divided from the AAL-90 template [49], was adopted. The parcellation scheme of the AAL-90 and AAL-1024 was presented in Figure S1. The functional connectivity matrix acquisition procedures were repeated for high parcellation scales. Then we obtained two correlation matrices (90×90 & 1024×1024) for each subject at the low and the high resolution parcellation respectively. A flowchart for the construction of resting-state functional brain network is shown in Figure 1A. These matrices served as the input for revealing the global topological organization of brain networks. We assessed the matrices at 11 different sparsity levels from 10% to 25%, at intervals of 1.5%. For a given network *G* with *N* nodes, sparsity (*S*) is defined as ratio of the actual edge number (*K*) to the maximum possible edge number [i.e., *N*(*N*-1)/2] in the network. Through choosing a threshold that preserves a specified percentage of the strongest edges in the network, it is possible to compare the topological organization among different groups.

### 4.4 Graph Theoretical Analysis

Graph theory is a natural framework for the mathematical representation of complex networks. Recently, graph theory has attracted considerable attention in brain network research because it provides a powerful way to quantitatively describe the segregation and integration of brain network from the perspective of the topological organization [5]. In this study, graph theoretical analysis was conducted with Brain Connectivity Toolbox [70].

#### 4.4.1 Global network metrics

Three network metrics, i.e., normalized clustering coefficient (*γ*), normalized shortest path length (*λ*), and small-worldness (*σ*), were adopted to reveal the global topological aberrations of the brain networks in AD and aMCI. The clustering coefficient is an index of the local inter-connectedness of the network, whereas the characteristic path length is an indicator of its overall connectedness [15]. Optimal brain functioning requires a balance between local specialization and global integration [4], [71]. For a given network *G* with *N* nodes, the clustering coefficient *C_i_* of a node *i* is defined as the ratio of the number of existing edges between its neighbor vertices to the number of maximum possible edges between the neighbor vertices:
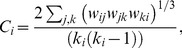
(1)where *k_i_* is the number of edges connecting node *i*, and *w_ij_* is the edge weight (i.e., correlation coefficient) between node *i* and node *j*. The clustering coefficient *C_w_* of the network was calculated as the mean of the clustering coefficients *C_i_* of all the nodes in the network. In network, a path between node *i* and node *j* refers to an edge that directly connects them or a sequence of edges that link them through other nodes. Then the shortest path length between node *i* and node *j* is defined as the minimum one of the sum of the edge lengths along all possible paths. Further, the shortest path length *L_w_* of a weighted graph was defined as the mean of the shortest path length of all pairs of nodes. In the present study, the reciprocal of the edge weight (1/*w_ij_*) was denoted as the length of an edge. To examine the small-world properties, the normalized weighted clustering coefficient 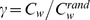 and the normalized shortest path length 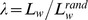 of brain networks were computed, in which 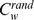 and 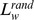 denote the average weighted clustering coefficient and the average shortest path length of an ensemble of 100 surrogate random networks. These random networks were derived from the original brain network by randomly rewiring the edges between nodes while preserving the weight distribution [72]. These two metrics can be unified as one metric, called small-worldness, i.e., *σ* = *γ*/*λ*. A real network is considered as small-world network if it meets the criteria: 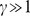 and *λ*≈1 [71].

#### 4.4.2 Regional nodal characteristics

In the current study, regional nodal characteristics were assessed via node betweenness. The betweenness centrality *bc_i_* of a node *i* is defined as the number of shortest paths between pairs of other nodes that pass through the node [73]. We calculated the normalized betweenness as 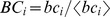, where 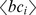 was the average betweenness of all nodes. Hence, the *BC_i_* captures the influence of a node over information flow between other nodes in the network. We then averaged the normalized betweenness across the sparsity range (10%<*S*<25%) to obtain the salient regional nodal characteristics [74].

#### 4.4.3 Modularity and regional role

Modularity is a fundamental concept in systems neuroscience, referring to the formation of local modules that nodes in the same module are densely connected to each other while nodes in different modules are sparsely connected [23]. Several algorithms have been proposed to quantify the partition in terms of module separation, i.e., how well a partition differentiates subsets of nodes tightly connected [75]. The modularity value *Q* of a network *G* for a given partition can be quantified as the proportion of *G*’s edges that fall within modules, subtracted by the proportion that would be expected due to random chance alone:
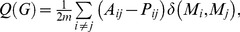
(2)where *m* is the total number of edges; *A_ij_* = 1 if an edge links node *i* and *j*; 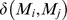 is 1 if node *i* and *j* are in the same module and 0 otherwise (ensure that only intra-modular edges are added to the sum), and *P_ij_* is the probability that there exist an edge between node *i* and node *j*, given a random network comparable to *G* [23]. The value of *P_ij_* could be estimated by 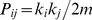, where *k_i_* is the total number of edges connecting node *i*. A strongly modular network has modularity value close to 1 while a network without modular organization has modularity value close to 0. It is generally accepted that maximal values of *Q*≥0.3 are indicative of non-random community structure. The spectral algorithm was adopted here for community detection [39].

In the current study, the community structure for the group averaged functional brain network were estimated at low parcellation scale (90×90) for the three groups respectively at a specific sparsity threshold 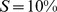 [20]. With this threshold, we can capture the network backbone underlying the modular organization of the most sparse yet maintain the connectedness (the ability for every node to reach other node in the network) for all the three groups [19] (see Figure S2). To further classify the nodes according to their topological functions in the network, two additional metrics, i.e., the within-module betweenness centrality (*sBC_i_*) and participation coefficient (*PC_i_*), were employed [19], [76], [77]. The *sBC_i_* is the betweenness centrality obtained within the module where node *i* belongs. *PC_i_* measures the inter-module connectivity of a node *i*:
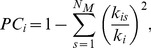
(3)where *k_i_* is the total number of edges linking to node *i*, *k_is_* is the number of edges linking node *i* to other nodes in the module *s*, and *N_M_* is the total number of modules. *PC_i_* will be close to 1 if node *i* has a homogeneous connection distribution with all the modules and 0 if it is linked exclusively to other nodes in its own module. A node could then be defined as modular hub if *sBC* > mean + S.D. and as a non-hub otherwise. In terms of *PC*, the hub node could be further characterized as connector hubs if *PC*>0.4, and otherwise as provincial hub. For non-hub node, node with *PC*>0.4 corresponds to connector node and node with *PC*≤0.4 is classified as provincial node [19], [45]. In addition to discrepancies in the composition and numbers of modules, we further investigate the topological roles of the modules in the brain networks. Connector coefficient of each module was calculated as the ratio between the number of connector nodes and the number of all nodes. The connector-module could then be defined as the module that has a high connector coefficient (connector coefficient 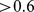) and a high ratio of intermodule connections (>1/number of modules) [19], [20].

### 4.5 Relationship between Functional Connectivity and Structural Connectivity

As a neurodegenerative disease, it has been widely accepted that AD is an anomaly of anatomic and/or functional connectivity [8], [37]. How the function-structural relationship varies as the progression of AD, however, is only beginning to be revealed. In the current study, structural connectivity (SC) was measured using diffusion tensor imaging (DTI). DTI fiber tractography is a direct way to depict the SC of brain network, by providing putative bundle pathways of macroscopic white matter fibers linking cortical areas. SC matrix was constructed with a toolbox for analyzing brain diffusion images [78]. A detailed flowchart for the SC network construction was shown in Figure S3. The nodes of the structural network were taken in a similar manner as the nodes in the functional network to enable the following analysis between the two types of networks. The relationship between the strength of FC and SC were firstly assessed in the three groups through examining the probability densities of FC when SC is present or absent [26]. We expect to observe a more robust relationship between the strength of FC and SC when direct SC is present – the mean value of the FC distribution should be significantly higher than 0. To further quantify the relationship between FC and SC and investigate the alternation of FC-SC coupling in AD and aMCI, we estimated the correlation coefficient of all nonzero entries of the SC matrix and their functional counterparts selected from FC matrix in each group (Figure 1B). This resulted in a single FC-SC coupling metric for each of the subjects. In order to obtain the refined probability densities of FC, the FC-SC relationship was estimated only at high-resolution (1024×1024) in the current study.

### 4.6 Statistical Analysis

A one-way analysis of variance (ANOVA) model comprising *Group* (AD, aMCI, & NC) as a between-subject factor was employed in the present study. Such model was adopted for global network metrics (i.e., *γ*, *λ*, and *σ*) at a specific sparsity threshold of *S* = 10%; and integrated network metrics (i.e., *I_γ_*, *I_λ_*, and *I_σ_*) which corresponded to the areas of curves within the sparsity range of 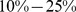 [6]. If main group effects were significant by ANOVA, between-group differences of network metrics were then determined by *post hoc* least significant difference (LSD) analysis. For the investigation of the nodal betweenness, ANOVA was performed to reveal the significant difference of the nodal centrality among the three groups. Bivariate association between the network parameters and clinical diagnostic results were assessed with Pearson’s correlations. A value of *p*<0.05 was considered significant. Note that the statistical results of the multiple ANOVAs entailed for the regional nodal analysis were considered as significant if *p*<0.05 (uncorrected) and were reported as exploratory results in nature. To investigate the influence of AD on the FC-SC coupling, a one-way ANOVA was conducted. All analyses were performed using SPSS (version 17.0, IBM, Armonk, New York).

## Supporting Information

Figure S1 **Two parcellation scales, (A) AAL-90 and (B) AAL-1024, were overlaid on the brain surface at the medium view.**
(TIF)

Figure S2 **The size of the largest connected component of the functional brain networks for AD, aMCI and NC groups as a function of sparsity threshold.** The largest connected component increases with the increment of sparsity. The black vertical line indicates the sparsity for a fully connected network among all the three groups.
(TIF)

Figure S3 **The flowchart of the structural brain network construction.** (1) The rigid coregistration from structural T1-weighted image (B) to the corresponding b0 image (C) through an affine transformation was initially performed. (2) A nonlinear transformation T was then obtained when registered the T1-weighted image (B) to the ICBM152 template in the Montreal Neurological Institute (MNI) space (A). (3) The inverse of the transformation (T^−1^) was applied to the high-resolution AAL template (D) to generate the corresponding subject-specific AAL mask (E). (4) The DTI (F) was constructed from the diffusion weighted images (C). (5) White matter fiber (G) reconstruction in the whole brain was performed using fiber assignment by continuous tracking (FACT) algorithm. The weighted network (SC network) of each subject was created by computing the fiber numbers that connected each pair of brain regions.
(TIF)

Table S1 Abbreviations of cortical regions of automated anatomical labeling (AAL-90). The brain regions were defined in terms of a prior template of an automated anatomical labeling (AAL) atlas defined by Tzourio-Mazoyer et al. 2002 [66]. Odd number index for left hemisphere and even number in the right hemisphere.
(DOCX)

Table S2 Comparisons of the integrated global networks measures over the sparsity range of 10%–25% among the AD, aMCI and NC groups. Values are represented as the mean (S.D.). For each participant, two functional connectivity networks were obtained via different parcellation scales (low: 90×90, and high: 1024×1024). The comparisons of the integrated global network measures among the three groups (AD, aMCI, & NC) were estimated using ANOVA. The bivariate correlation of the network metrics with MMSE was performed using Pearson’s correlation. ^†^ *Post hoc* test showed significant group difference between control *v.s.* AD; *Post hoc* pairwise comparison was performed through Fisher’s least significant difference (LSD) and *p*<0.05 was considered significant. **Bold** data indicates statistical significance.
(DOCX)

Table S3 Neuropsychological data between NC and aMCI patients. NC: normal control subjects; aMCI: amnestic mild cognitive impairment patients; AVLT: auditory verbal learning task (Chinese version) [79]; SCWT: Stroop color-word test [80]; CDT: clock-drawing test; AVF: animal verbal frequency. *p*<0.05 was considered significant, and **bold** data indicated statistical significance. ^b^ Missing data of 1 subject in aMCI group.
(DOCX)
