# Supplementary figures and images for: Disrupted Functional Brain Connectivity and Its Association to Structural Connectivity in Amnestic Mild Cognitive Impairment and Alzheimer’s Disease
Source: PLoS One. 2014 May 7;9(5):e96505. doi: 10.1371/journal.pone.0096505 (PMC4013022; doi:10.1371/journal.pone.0096505)

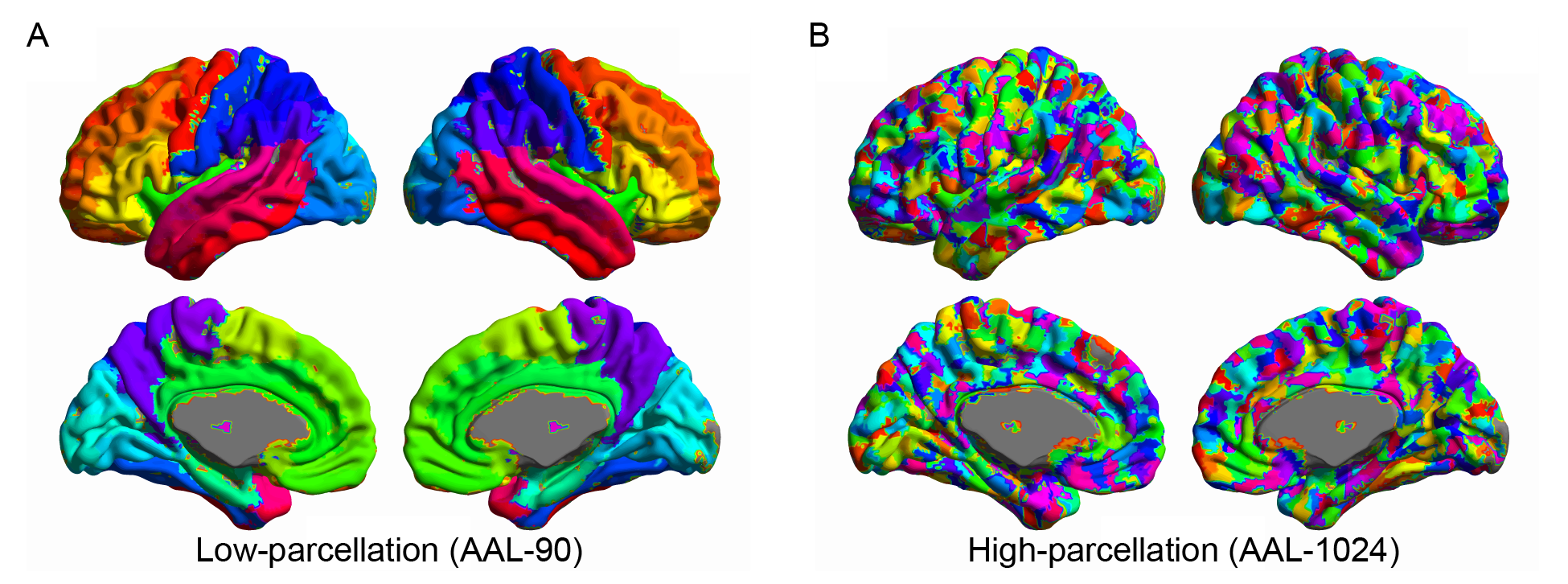

Supplement: Figure S1 — Two parcellation scales, (A) AAL-90 and (B) AAL-1024, were overlaid on the brain surface at the medium view. (TIF) [file pone.0096505.s001.tif]

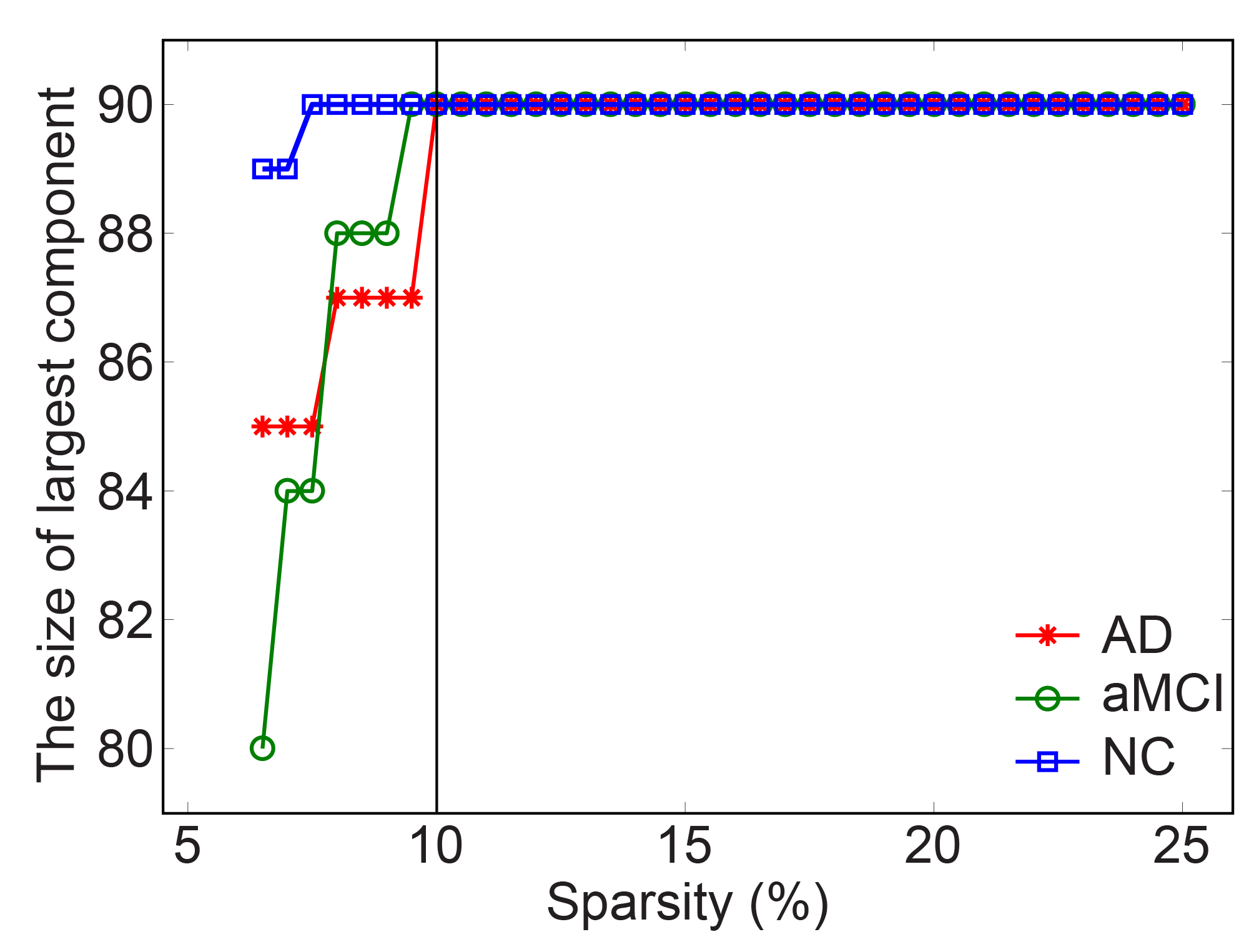

Supplement: Figure S2 — The size of the largest connected component of the functional brain networks for AD, aMCI and NC groups as a function of sparsity threshold. The largest connected component increases with the increment of sparsity. The black vertical line indicates the sparsity for a fully connected network among all the three groups. (TIF) [file pone.0096505.s002.tif]

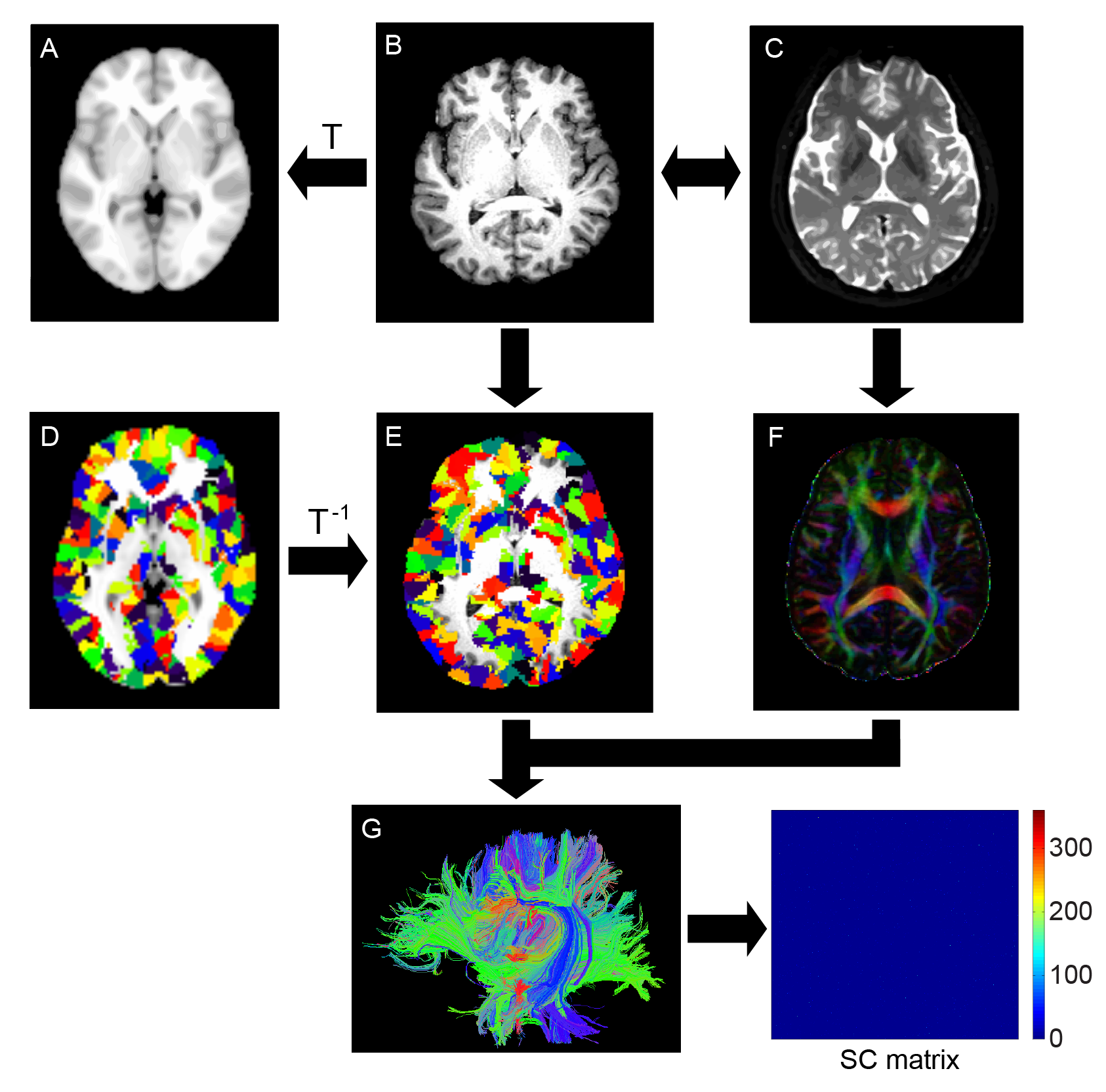

Supplement: Figure S3 — The flowchart of the structural brain network construction. (1) The rigid coregistration from structural T1-weighted image (B) to the corresponding b0 image (C) through an affine transformation was initially performed. (2) A nonlinear transformation T was then obtained when registered the T1-weighted image (B) to the ICBM152 template in the Montreal Neurological Institute (MNI) space (A). (3) The inverse of the transformation (T−1) was applied to the high-resolution AAL template (D) to generate the corresponding subject-specific AAL mask (E). (4) The DTI (F) was constructed from the diffusion weighted images (C). (5) White matter fiber (G) reconstruction in the whole brain was performed using fiber assignment by continuous tracking (FACT) algorithm. The weighted network (SC network) of each subject was created by computing the fiber numbers that connected each pair of brain regions. (TIF) [file pone.0096505.s003.tif]
