# Supplementary material for: Disrupted Functional Brain Connectivity and Its Association to Structural Connectivity in Amnestic Mild Cognitive Impairment and Alzheimer’s Disease
Source: PLoS One. 2014 May 7;9(5):e96505. doi: 10.1371/journal.pone.0096505 (PMC4013022; doi:10.1371/journal.pone.0096505)
Supplement: Table S1 — Abbreviations of cortical regions of automated anatomical labeling (AAL-90). The brain regions were defined in terms of a prior template of an automated anatomical labeling (AAL) atlas defined by Tzourio-Mazoyer et al. 2002 [66]. Odd number index for left hemisphere and even number in the right hemisphere. (DOCX) [file pone.0096505.s004.docx]

| **Index** | **Cortical regions** | **Abbreviation** | **Class** |
| --- | --- | --- | --- |
| (1,2) | Precentral gyrus | PreCG | Primary |
| (3,4) | Superior frontal gyrus, dorsolateral | SFGdor | Association |
| (5,6) | Superior frontal gyrus, orbital part | ORBsup | Paralimbic |
| (7,8) | Middle frontal gyrus | MFG | Association |
| (9,10) | Middle frontal gyrus, orbital part | ORBmid | Paralimbic |
| (11,12) | Inferior frontal gyrus, opercular part | IFGoperc | Association |
| (13,14) | Inferior frontal gyrus, triangular part | IFGtriang | Association |
| (15,16) | Inferior frontal gyrus, orbital part | ORBinf | Paralimbic |
| (17,18) | Rolandic operculum | ROL | Association |
| (19,20) | Supplementary motor area | SMA | Association |
| (21,22) | Olfactory cortex | OLF | Paralimbic |
| (23,24) | Superior frontal gyrus, medial | SFGmed | Association |
| (25,26) | Superior frontal gyrus, medial orbital | ORBsupmed | Paralimbic |
| (27,28) | Gyrus rectus | REC | Paralimbic |
| (29,30) | Insula | INS | Paralimbic |
| (31,32) | Anterior cingulate and paracingulate gyri | ACG | Paralimbic |
| (33,34) | Median cingulate and paracingulate gyri | DCG | Paralimbic |
| (35,36) | Posterior cingulate gyrus | PCG | Paralimbic |
| (37,38) | Hippocampus | HIP | Subcortical |
| (39,40) | Parahippocampal gyrus | PHG | Paralimbic |
| (41,42) | Amygdala | AMYG | Paralimbic |
| (43,44) | Calcarine fissure and surrounding cortex | CAL | Primary |
| (45,46) | Cuneus | CUN | Association |
| (47,48) | Lingual gyrus | LING | Association |
| (49,50) | Superior occipital gyrus | SOG | Association |
| (51,52) | Middle occipital gyrus | MOG | Association |
| (53,54) | Inferior occipital gyrus | IOG | Association |
| (55,56) | Fusiform gyrus | FFG | Association |
| (57,58) | Postcentral gyrus | PoCG | Primary |
| (59,60) | Superior parietal gyrus | SPG | Association |
| (61,62) | Inferior parietal, but supramarginal and angular gyri | IPL | Association |
| (63,64) | Supramarginal gyrus | SMG | Association |
| (65,66) | Angular gyrus | ANG | Association |
| (67,68) | Precuneus | PCUN | Association |
| (69,70) | Paracentral lobule | PCL | Association |
| (71,72) | Caudate nucleus | CAU | Subcortical |
| (73,74) | Lenticular nucleus, putamen | PUT | Subcortical |
| (75,76) | Lenticular nucleus, pallidium | PAL | Subcortical |
| (77,78) | Thalamus | THA | Subcortical |
| (79,80) | Heschl gyrus | HES | Primary |
| (81,82) | Superior temporal gyrus | STG | Association |
| (83,84) | Temporal pole: superior temporal gyrus | TPOsup | Paralimbic |
| (85,86) | Middle temporal gyrus | MTG | Association |
| (87,88) | Temporal pole: middle temporal gyrus | TPOmid | Paralimbic |
| (89,90) | Inferior temporal gyrus | ITG | Association |
