# Supplementary material for: Disrupted Functional Brain Connectivity and Its Association to Structural Connectivity in Amnestic Mild Cognitive Impairment and Alzheimer’s Disease
Source: PLoS One. 2014 May 7;9(5):e96505. doi: 10.1371/journal.pone.0096505 (PMC4013022; doi:10.1371/journal.pone.0096505)
Supplement: Table S2 — Comparisons of the integrated global networks measures over the sparsity range of 10%–25% among the AD, aMCI and NC groups. Values are represented as the mean (S.D.). For each participant, two functional connectivity networks were obtained via different parcellation scales (low: 90×90, and high: 1024×1024). The comparisons of the integrated global network measures among the three groups (AD, aMCI, & NC) were estimated using ANOVA. The bivariate correlation of the network metrics with MMSE was performed using Pearson’s correlation. † Post hoc test showed significant group difference between control v.s. AD; Post hoc pairwise comparison was performed through Fisher’s least significant difference (LSD) and p<0.05 was considered significant. Bold data indicates statistical significance. (DOCX) [file pone.0096505.s005.docx]

|  |  |  |  | ANOVA | | Correlation with MMSE | |
| --- | --- | --- | --- | --- | --- | --- | --- |
|  | AD | aMCI | NC | F value | *p* value | *r* value | *p* value |
| Low parcellation (90-ROI) | |  |  |  |  |  |  |
| *I_γ_* | 0.279 (0.032) | 0.308 (0.034) | 0.316 (0.046) | 3.403 | **0.044**^†^ | 0.354 | **0.023** |
| *I_λ_* | 0.183 (0.008) | 0.179 (0.006) | 0.179 (0.006) | 2.232 | 0.121 | -0.247 | 0.119 |
| *I_σ_* | 0.228 (0.031) | 0.258 (0.026) | 0.264 (0.035) | 4.994 | **0.012**^†^ | 0.403 | **0.009** |
| High parcellation (1024-ROI) | |  |  |  |  |  |  |
| *I_γ_* | 0.256 (0.191) | 0.269 (0.023) | 0.277 (0.021) | 3.243 | **0.050**^†^ | 0.395 | **0.011** |
| *I_λ_* | 0.187 (0.008) | 0.189 (0.005) | 0.186 (0.006) | 0.825 | 0.446 | 0.048 | 0.768 |
| *I_σ_* | 0.205 (0.017) | 0.214 (0.020) | 0.224 (0.017) | 3.514 | **0.040**^†^ | 0.348 | **0.026** |
