# Supplementary material for: Disrupted Functional Brain Connectivity and Its Association to Structural Connectivity in Amnestic Mild Cognitive Impairment and Alzheimer’s Disease
Source: PLoS One. 2014 May 7;9(5):e96505. doi: 10.1371/journal.pone.0096505 (PMC4013022; doi:10.1371/journal.pone.0096505)
Supplement: Table S3 — Neuropsychological data between NC and aMCI patients. NC: normal control subjects; aMCI: amnestic mild cognitive impairment patients; AVLT: auditory verbal learning task (Chinese version) [79]; SCWT: Stroop color-word test [80]; CDT: clock-drawing test; AVF: animal verbal frequency. p<0.05 was considered significant, and bold data indicated statistical significance. b Missing data of 1 subject in aMCI group. (DOCX) [file pone.0096505.s006.docx]

|  | NC (n = 14) | | aMCI (n = 15) | |  |
| --- | --- | --- | --- | --- | --- |
|  | mean | S.D. | mean | S.D. | *p*-value |
| AVLT immediate recall | 16.36 | 4.24 | 8.13 | 4.29 | **<0.0001** |
| AVLT delayed recall | 5.64 | 1.45 | 1.20 | 1.21 | **<0.0001** |
| SCWT, word | 28.79 | 5.73 | 33.67 | 7.95 | 0.1523 |
| SCWT, color ^b^ | 39.79 | 9.37 | 51.21 | 12.19 | **0.0099** |
| SCWT, word-color ^b^ | 69.86 | 15.37 | 106.71 | 40.19 | **0.0036** |
| CDT | 4.43 | 0.65 | 3.73 | 1.39 | 0.0989 |
| AVF | 16.57 | 3.44 | 12.33 | 3.39 | **0.0025** |
